# Host Plant Adaptation in *Drosophila mettleri* Populations

**DOI:** 10.1371/journal.pone.0034008

**Published:** 2012-04-06

**Authors:** Sergio Castrezana, Jeremy M. Bono

**Affiliations:** 1 Department of Ecology and Evolutionary Biology, University of Arizona, Tucson, Arizona, United States of America; 2 Department of Biology, University of Colorado Colorado Springs, Colorado Springs, Colorado, United States of America; American Museum of Natural History, United States of America

## Abstract

The process of local adaptation creates diversity among allopatric populations, and may eventually lead to speciation. Plant-feeding insect populations that specialize on different host species provide an excellent opportunity to evaluate the causes of ecological specialization and the subsequent consequences for diversity. In this study, we used geographically separated *Drosophila mettleri* populations that specialize on different host cacti to examine oviposition preference for and larval performance on an array of natural and non-natural hosts (eight total). We found evidence of local adaptation in performance on saguaro cactus (*Carnegiea gigantea*) for populations that are typically associated with this host, and to chemically divergent prickly pear species (*Opuntia spp.*) in a genetically isolated population on Santa Catalina Island. Moreover, each population exhibited reduced performance on the alternative host. This finding is consistent with trade-offs associated with adaptation to these chemically divergent hosts, although we also discuss alternative explanations for this pattern. For oviposition preference, Santa Catalina Island flies were more likely to oviposit on some prickly pear species, but all populations readily laid eggs on saguaro. Experiments with non-natural hosts suggest that factors such as ecological opportunity may play a more important role than host plant chemistry in explaining the lack of natural associations with some hosts.

## Introduction

Ecological specialization generates diversity within species, and may ultimately lead to speciation [1]–[3]. This is particularly likely when ecological divergence occurs among allopatric populations, as restricted gene flow creates favorable conditions for local adaptation and genetic differentiation. Local adaptation is most probable when ecological environments experienced by different populations are highly divergent, but gene flow or small population size may limit adaptive divergence [4], [5]. Local adaptation may also result in trade-offs, whereby adaptation to the local environment leads to poor performance in alternative environments due to antagonistic pleiotropy [1], [2]. Theoretical models indicate that while such trade-offs may facilitate specialization and subsequent speciation, they are not required for specialization to evolve [6].

Plant-breeding insects provide an interesting opportunity to identify the causes of specialization and the subsequent consequences for diversification [7]. While many plant-breeding insect species are strict specialists, even polyphagous species often exhibit some degree of host specialization at the population level, as individuals in different locations may shift to novel hosts [8]. Such shifts may occur because newly encountered plants are chemically superior for adult feeding and larval development, because they represent a more predictable resource, or simply because favored hosts are not available. Regardless, host shifts should be accompanied by selection for increased preference for and performance on the new host, including physiological adaptations to host plant chemistry.

*Drosophila mettleri* is one of several *Drosophila* species from arid regions of the southwestern United States and northwestern Mexico that uses decomposing cactus as a breeding substrate. Associations among these *Drosophila* and their host plants appear to be determined in large part by physiological adaptations to plant chemistry as the hosts contain various toxic allelochemicals that are not tolerable to all the *Drosophila* species [9]. *Drosophila mettleri* is unique among the desert *Drosophila* species because females oviposit in soil that is soaked with rotting cactus exudates rather than in the necrotic cactus tissue. This behavior has important consequences for developing larvae, as high evaporation rates in the arid desert environment can concentrate toxins in soaked soil to levels up to nearly 30 times that found in rotting tissue [10]. Although highly toxic, the soil environment is free of competitors found within the rotting tissue, including other *Drosophila* and other arthropod species.

*Drosophila mettleri* is associated with columnar cactus hosts in the core parts of its geographic range. Populations in the northern Mexican state of Sonora and Arizona, USA are associated mainly with saguaro cactus (*Carnegiea gigantea*), though adults and larvae are commonly collected from cardón cactus (*Pachycereus pringleii)* in a few isolated patches in northern Mexico where this species occurs. In addition, adults and larvae are occasionally collected from hecho cactus (*Pachycereus pecten-aboriginum*), senita cactus *(Lophocereus schotti)*, barrel cactus, (*Ferocactus cylindraceus*), (S. Castrezana, personal observations), and organ pipe cactus (*Stenocereus thurberi*) [11] in this area. With the exception of saguaro, all of these hosts are present on the Baja California peninsula, but associations with cardón are by far most frequent. A geographically isolated population also occurs on Santa Catalina Island off the coast of southern California, where flies use prickly pear cactus (*Opuntia* spp.), as none of the columnar cacti are available in this location. Genetic evidence indicates that this population is derived and genetically differentiated from other *D. mettleri* populations [12]–[14]. Although laboratory studies have demonstrated that *D. mettleri* larvae can be reared from several host species [10], [11], [15], there has been no systematic effort to examine population level variation in adult preference for and larval performance on different hosts.

In this study, we use *D. mettleri* collected from four different geographic localities to assess preference for and performance on eight natural and non-natural cactus host species. Our primary objective was to investigate whether populations that primarily specialize on saguaro (northern Mexico and Arizona, USA) and prickly pear (Santa Catalina Island) show evidence of local adaptation to these hosts, and whether results are consistent with the possibility of trade-offs. Following Hereford [4], we consider higher fitness of flies on their native host relative to flies from other populations on this host as evidence of local adaptation. If locally adapted populations also show reduced fitness on alternative host plants, this would be consistent with trade-offs [4], though subsequent tests would be necessary to demonstrate that anatagonistic pleiotropy is the underlying cause. We predicted that local adaptation would be particularly likely on Santa Catalina Island given the strong genetic isolation of this population [12]–[14] and differences in plant chemistry between prickly and the other main hosts [16]–[18]. While we likewise predicted that saguaro specialist populations could show evidence of local adaptation, this possibility might be mitigated by gene flow from populations on the Baja peninsula, where saguaro is absent. In addition to this main objective, we also examine whether preference/performance data can explain the relative rarity of associations with some naturally available hosts, and whether taxonomic relationships and/or chemical similarity between hosts are predictive of relative levels of preference and performance.

## Methods

### Fly collection and handling

**Figure 1:**
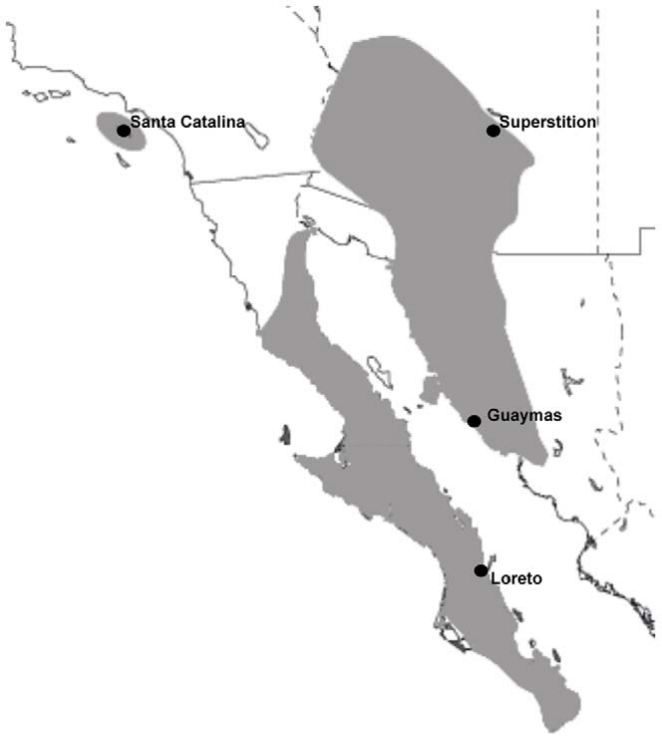
Geographic distribution of *Drosophila mettleri*. The geographic distribution of *Drosophila mettleri* is shaded in gray and the four localities where flies were collected for these experiments are indicated.

We used *D. mettleri* cultures initiated from multi-female collections (N>40) from four localities: (1) Superstition Mountains, Arizona, USA, (2) Guaymas, Sonora, Mexico, (3) Loreto, South Baja California, Mexico, and (4) Santa Catalina Island, off the coast of southern California, USA, (Fig. 1). Flies from Mexican populations were imported under permit #69541 issued by the United States Department of Agriculture, while flies from Santa Catalina Island were collected with the approval of the Catalina Island Conservancy. No special permits were required for Arizona collections; flies are not protected or endangered and collections were not made on protected lands.

Virgin flies used in our experiments were separated by sex under CO_2_ and maintained on a standard cornmeal food (5–10 flies/vial) at 25°C for seven days.

### Cactus collection and handling

We included eight species of cacti in our experiments. Five of the species were columnar cacti from the Pachycereeae tribe: saguaro, senita, hecho, organ pipe, and agria (*Stenocereus gummosus*). Three of these, (saguaro, senita, and hecho), contain toxic alkaloids, but the identity, complexity, and concentration of these compounds varies among them [16], [17], [19]. For logistical reasons, we were unable to include the main host on the Baja peninsula (cardón). Cardón is most closely related to hecho [20] and is considered to be relatively similar chemically to *D. mettleri's* other main host, saguaro [9], [21]. The two species in the genus *Stenocereus* (organ pipe and agria) do not contain alkaloids, but are rich in other toxic compounds such as triterpene glycosides, medium chain fatty acids, and sterol diols [16], [17]. Our assays also included three different *Opuntia* species: tulip prickly pear (*O.phaeacantha*), mission prickly pear (*O. ficus-indica*), and Engelmann's prickly pear (*O. engelmannii*), the first two of which are found on Santa Catalina Island. Although less is known about the plant chemistry of the *Opuntia*, both tulip prickly pear and mission prickly pear are known to contain alkaloids [18], [22]. To our knowledge, no information is available on the chemical composition of Engelmann's prickly pear, but it is closely related to tulip prickly pear [23]. A screen of alkaloids from several *Opuntia* species, including tulip prickly pear, revealed that most were novel compared to any previously identified cactus alkaloids. This, in addition to other chemical differences between prickly pear species and columnar cacti [16], [17], suggests that *D. mettleri's* prickly pear hosts are chemically divergent relative to the other main hosts (saguaro and cardón). Summary information on plant chemistry and availability of each cactus species at collecting sites is given in Table 1. All cactus collections were made under permit #P588-070826-001 issued by the United States Department of Agriculture and CITES permit #35032.

**Table 1:** Population availability of host plants used in experiments and information on the main toxic compounds present in each species (if known).

Cactus species	Common name	Plant tribe	Population availability	Toxic components (% dry weight if known) [Ref.]
*Carnegiea gigantea*	Saguaro	Pachycereeae	Superstition	Alkaloids (1–3%) [16], [17]
			Guaymas	
*Pachycereus pectin-aboriginum*	Hecho	Pachycereeae	Guaymas	Alkaloids [19]
			Loreto	
*Lophocereus schottii*	Senita	Pachycereeae	Guaymas	Alkaloids (15–20%) [16], [17]
			Loreto	
*Stenocereus thurberi*	Organ pipe	Pachyreeae	Guaymas	Triterpene glycosides (20–30%)
			Loreto	Sterol diols
				Medium chain fatty acids [16], [17]
*Stenocereus gummosus*	Agria	Pachycereeae	Loreto	Triterpene glycosides (30–40%)
				Sterol diols
				Medium chain fatty acids [16], [17]
*Opuntia ficus-indica*	Mission prickly pear	Opuntieae	Superstition	Alkaloids [22]
			Guaymas	
			Catalina	
*Opuntia engelmannii*	Engelmann's prickly pear	Opuntieae	Superstition	?
			Loreto	
*Opuntia phaeacantha*	Tulip prickly pear	Opuntieae	Superstition	Alkaloids [18]
			Guaymas	
			Loreto	
			Catalina	

To prepare cactus for preference-performance assays, we boiled approximately 400 g of tissue from 25 different plants in three liters of distilled water for five minutes. We then blended the cactus tissue with two liters of the water and stored the solution at −10°C, until use in the experimental assays.

### Oviposition assays

To determine female oviposition preferences, we used a ‘no-choice’, or ‘acceptance test,’ where different females were given the opportunity to oviposit on the various cacti individually. Although preference also can be assessed through choice tests, we believe that a no-choice test most closely approximates the situation experienced by flies in nature because cactus rots are relatively uncommon, so females are likely to encounter hosts sequentially rather than simultaneously. We prepared oviposition substrates by adding approximately two milliliters of liquefied cactus tissue to each well of a 24-well tissue culture plate (Sarstedt Co.). In order to create an oviposition substrate that was as natural as possible for *D. mettleri*, we then added sterilized sand (0.05 mm round) until the surface was firm and moist. We perforated the culture plate lids using a craft glue-gun, and a craft stick was introduced into each well as a perching site for females. A recently mated female was added to each well of the culture plate and sealed with a cotton plug (N = 30 for each population/cactus combination). We removed females after 24 hours and counted eggs under a dissecting microscope. We calculated relative preferences for hosts based on comparisons of the average number of eggs laid on each host.

### Larval viability assay

We placed 60 *D. mettleri* male/female pairs into a three-gallon plexi-glass cage containing a petri dish filled with mashed banana and baker's yeast for six hours. When eggs hatched, larvae were removed within 12 hours and placed in groups of 10 into glass vials filled with liquefied cactus/sterilized soil (25 vials for each population/cactus combination). We added a craft stick to create a site for pupation. Emerging adults were collected daily, and comparisons of larval to adult viability were used as a measure of offspring performance.

### Data analysis

We conducted all statistical analyses using SAS (v. 9.2; SAS Institute Inc., Cary, NC, USA). Our analysis of female preference was complicated by the fact that some females did not oviposit at all, which resulted in a non normal distribution of residuals that could not be corrected by transformation. Consequently, we divided our analysis into two parts. First, we analyzed whether there were differences in the proportion of females accepting a particular cactus (acceptance = laid at least one egg), and second, for those females that did oviposit, we analyzed whether there were differences in the number of eggs laid on different cacti. For the first analysis, we used the glimmix procedure to perform logistic regression (specified by the “data = binary” and “link = logit” options) with a model that included population, cactus, and a population x cactus interaction term as fixed effects. Unfortunately, no females for some population/cactus populations laid eggs (all populations on agria; Superstition, Guaymas, and Loreto on tulip prickly pear; Loreto on Engelmann's prickly pear). Inclusion of these data thus resulted in quasi-complete separation in the dataset, which can make the results of logistic regression unreliable. To alleviate this problem, we excluded these particular combinations from the analysis. Although these combinations were therefore not analyzed statistically, we reasonably conclude that females were more averse to ovipositing on these cacti than any of the others (i.e. preference was low). For the second analysis, we used a standard general linear model using proc glimmix with the identity link function. The dependent variable in this analysis was the number of eggs laid by a female (log-transformed), and explanatory variables included population, cactus, and a population x cactus interaction term (all fixed effects).

We analyzed larval to adult viability using the glimmix procedure, specifying the response as binary (survive = yes or no) and using the ‘logit’ link function. The model included population, cactus, the population x cactus interaction, and vial nested within population and cactus as a random effect. For all analyses we compared levels within a factor using the lsmeans statement with Tukey's adjustment for multiple testing. For significant interaction terms we were primarily interested in comparing simple effects, which we achieved by using the “testslice” option available in proc glimmix.

## Results

### Local adaptation: preference

**Figure 2:**
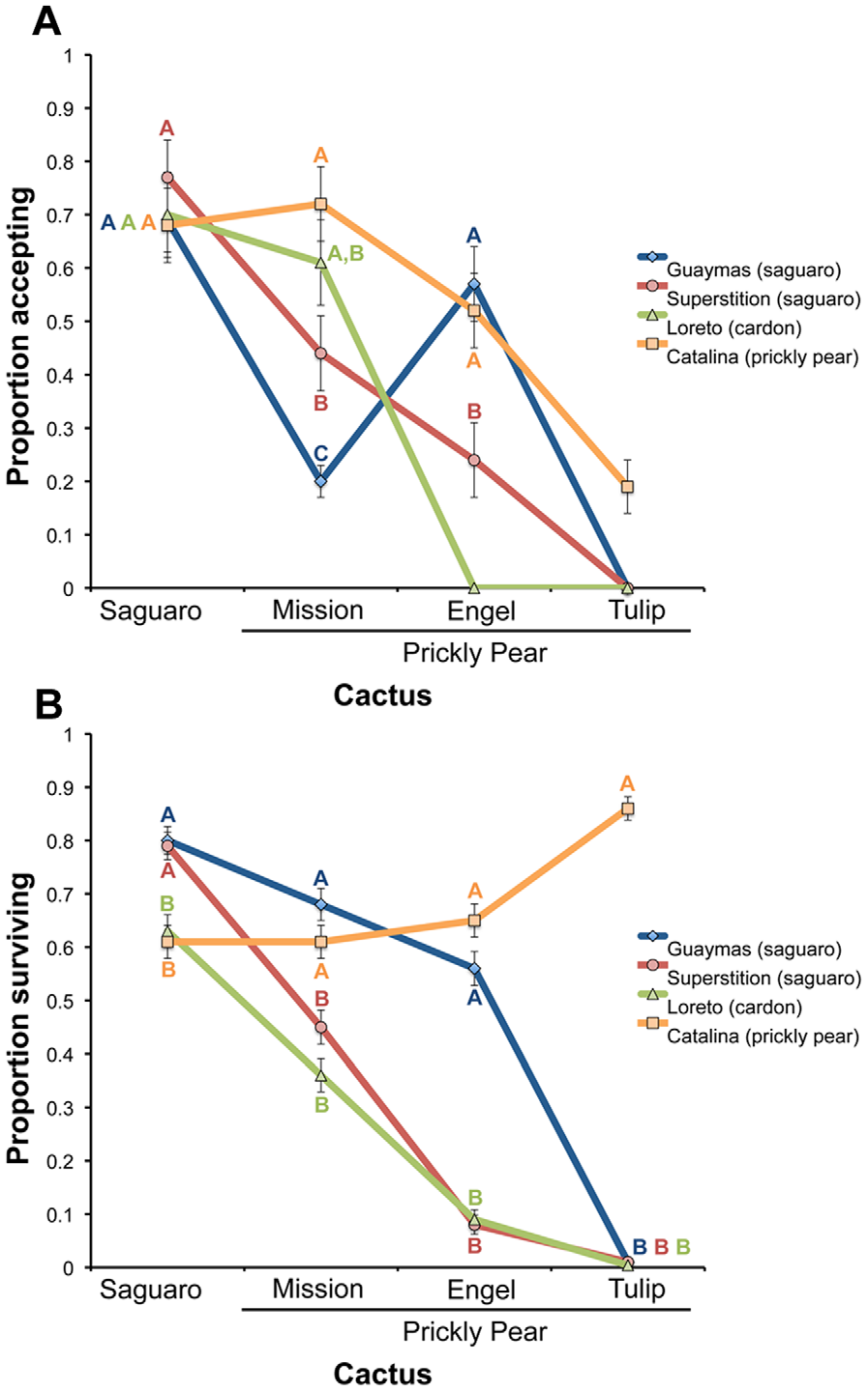
Local preference/performance adaptation. Least square means comparisons of (a) oviposition acceptance and (b) larval survival on saguaro and three species of prickly pear. Natural hosts for each population are given in parentheses in the key. For a given host plant, points under different letters were significantly different after Tukey's adjustment (α = 0.05). Points with no letter were not included in the statistical analysis (see methods).

Analysis of oviposition acceptance data revealed significant main effects and significant population x cactus interactions (Table 2). To evaluate evidence for local adaptation, we explored the nature of the population x cactus interaction by comparing populations within each cactus type. There was no evidence for local adaptation to saguaro in the Superstition or Guaymas populations, as this cactus was accepted at a similarly high rate in all populations (Figure 2a). There was, however, some evidence for local adaptation to prickly pear resources on Santa Catalina Island. Indeed, this was the only population willing to oviposit on tulip prickly pear (present on the island), albeit at a low rate, and acceptance was also relatively high for the other available host on the island, mission prickly pear, though this was not statistically distinguishable from the Loreto population (Figure 2a). For the number of eggs laid by females, both main effects and the population by cactus interaction were significant (Table 2). Further analysis of population comparisons suggests that, with a few exceptions, females laid about the same number on different hosts (Figure S1a). Thus, for these hosts, females appear to discriminate mainly by rejecting non-favored hosts altogether rather than adjusting the number of eggs laid.

**Table 2:** Model terms and their associated *P*-values from analyses using generalized linear models.

Response	Term	Numerator df, denominator df	*F*	*P*
Acceptance	Population	3, 1074	13.86	<0.0001
	Cactus	6, 1074	12.70	<0.0001
	Population*Cactus	14, 1074	7.84	<0.0001
Mean no. eggs laid	Population	3, 483	27.77	<0.0001
	Cactus	6, 483	20.28	<0.0001
	Population*Cactus	14, 483	5.83	<0.0001
Larval viability	Population	3, 768	55.94	<0.0001
	Cactus	7, 768	71.58	<0.0001
	Population*Cactus	21, 768	31.99	<0.0001

### Local adaptation: performance

Analysis of larval to adult viability indicated that there was a significant population x cactus interaction in addition to significant main effects of these two factors (Table 2). Population comparisons revealed evidence of local adaptation to saguaro and prickly pear by populations that primarily specialize on these hosts in nature. In fact, both the Guaymas and Superstition populations, which primarily use saguaro (though Guaymas also frequently uses limited patches of cardón), outperformed the other two populations on this host (Figure 2b), while there was a general trend for Santa Catalina Island flies to outperform the other three populations on the three *Opuntia* species (Figure 2b). This is particularly evident for larvae reared on tulip prickly pear, which showed nearly zero viability in the Superstition, Guaymas, and Loreto populations, but high viability (86%) in the Santa Catalina Island population. A similar trend was observed for larvae reared on Engelmann's prickly pear and mission prickly pear, except that there were no significant differences between the Santa Catalina Island and Guaymas populations, and the other two populations had at least low to moderate viability on these cacti (Figure 2b).

Performance data was also consistent with the possibility that trade-offs are associated with adaptation to saguaro and prickly pear. This is especially evident in comparisons between the Superstition and Santa Catalina Island populations, where each population had relatively higher performance on native host/s and reduced performance on the alternative host/s (Figure 2b). A similar trend was also observed for comparisons between Guaymas and Santa Catalina Island, except that the Guaymas population only showed reduced performance on one of the prickly pear species (Figure 2b).

### Preference/performance on non-natural hosts

**Figure 3:**
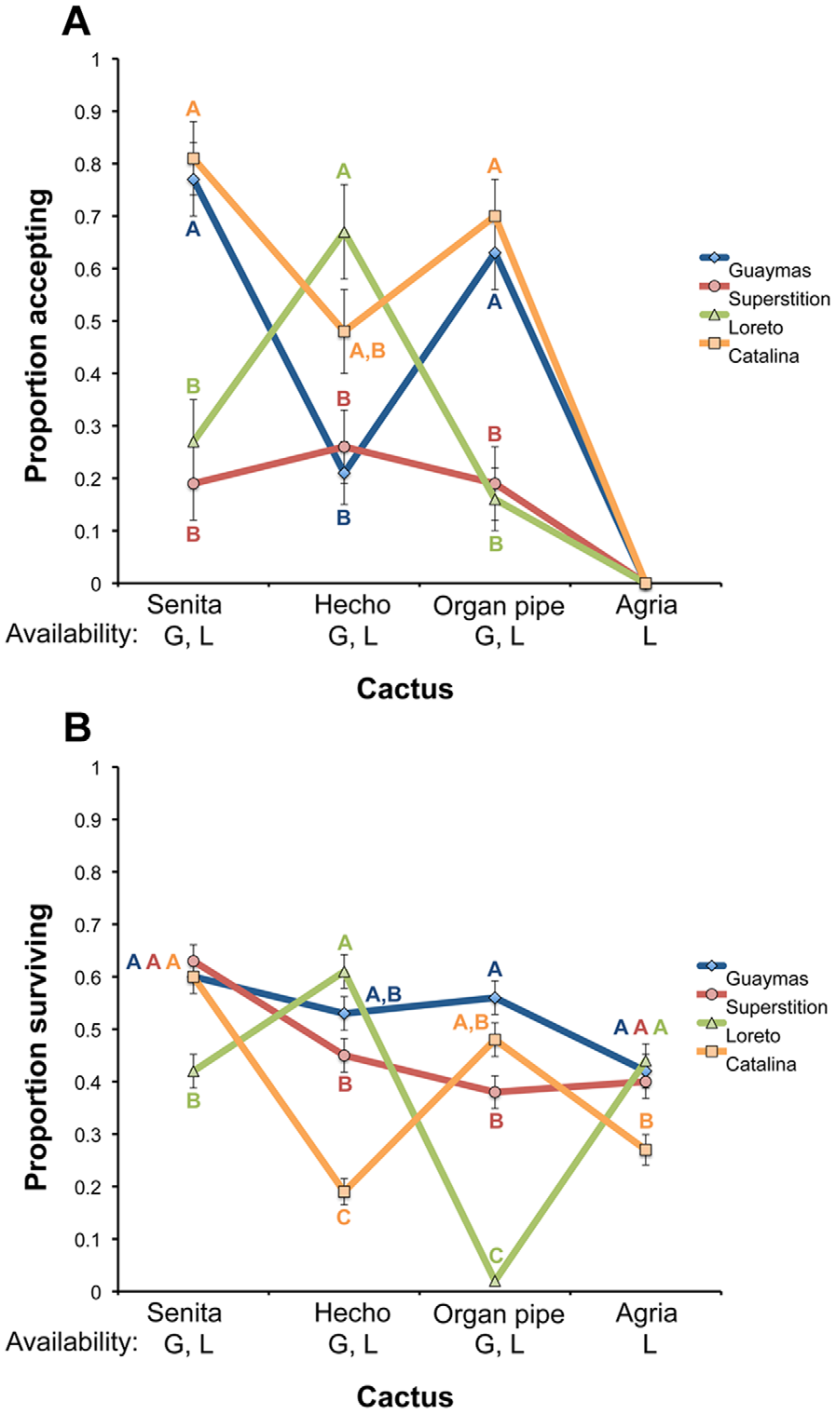
Preference/performance on non-natural hosts. Least square mean comparisons of (a) ovipostion acceptance and (b) larval survival on four cactus species rarely used as hosts in nature. Locations in which each cactus species is available are given at the bottom (G = Guaymas; S = Superstition; L = Loreto; C = Catalina). For a given host plant, points under different letters were significantly different after Tukey's adjustment (α = 0.05). Points with no letter were not included in the statistical analysis (see methods).

Acceptance of non-natural hosts was highly variable across populations, but, interestingly, females commonly oviposited on a wide range of hosts, even those from which larvae are rarely collected in nature (Figure 3a; Figure S1b). One exception to this is that females from all populations completely rejected agria cactus. Despite willingness to oviposit on a wide range of plants, in no case did preference for non-natural hosts exceed that for a natural host (Figure S2). Similarly, with a few exceptions, most populations had at least moderate viability on non-natural hosts (Figure 3b), though, again, performance was either equal or lower compared to performance on natural hosts (Figure S3).

## Discussion

The results of our preference/performance assays yield new insights into patterns of host plant adaptation in the *D. mettleri* study system, and how host plant chemistry and ecological opportunity combine to influence host plant associations in different geographic populations.

### Local adaptation

Analysis of performance data on saguaro and prickly pear revealed evidence of local adaptation for populations that primarily specialize on these hosts (i.e. Superstition and Guaymas for saguaro and Santa Catalina Island for prickly pear). For saguaro specialist populations this is true despite the possibility of migration between Baja and mainland Mexico [12]–[14], suggesting that divergent selection may be strong relative to the amount of gene flow between these locations. Although the toxic alkaloids in cardón and saguaro are similar in overall complexity and concentration [9], [21], our results indicate that differences may nonetheless be significant enough to constitute an important selective factor for traits involved in performance. In fact, earlier studies have suggested that *D. mettleri* detoxification enzymes might be highly substrate specific [24], [25], which could explain for why tolerance is not necessarily general, even across these closely related species.

A switch to chemically divergent hosts, coupled with strong geographic isolation has apparently driven local adaptation on Santa Catalina Island. In particular, the dramatically higher viability of larvae from this population on tulip prickly pear relative to flies from other populations suggests that Santa Catalina Island flies have acquired a unique ability to utilize this species, even though this host is also available throughout much of the rest of *D. metteri's* range. It is unclear what plant qualities contribute to the poor performance of most populations on this prickly pear species (and to a lesser extent the other two species), but the chemical composition of species in the genus *Opuntia* differs in many ways from the Pachycereeae. In addition to differences in lipid content, prickly pear species contain more free sugars, as sugars in the columnar cacti are generally tied up in more complex molecules [16], [17]. Moreover, a survey of the alkaloid content of several *Opuntia* species revealed that most contained alkaloids (including tulip prickly pear), almost all of which were novel when compared with known cactus alkaloids [18]. Although the general poor performance by flies from most populations on *Opuntia* could reflect reduced efficiency in utilizing nutritional resources, the almost complete lack of viability on tulip prickly pear and Engelmann's prickly pear (for some populations) seem more consistent with an inability to tolerate alkaloids or other toxic compounds.

Our results also demonstrate that adaptation to prickly pear is associated with reduced performance on saguaro (and vice versa), a finding consistent with the idea of trade-offs. Trade-offs are expected when genes involved in adaptation to a given environment have antagonistic pleiotropic effects in alternative environments [1], [2], [26]. In a recent review, Hereford [4] found that trade-offs are generally stronger when environments are more divergent. Given the chemical differences between prickly pear and saguaro, our results are consistent with this observation. The presence of trade-offs could also explain why populations on the mainland are not better adapted to prickly pear despite its availability, as negative effects on associations with the main host, saguaro, would be costly. Although our results are consistent with trade-offs, we do note that alternative explanations for our data are also plausible. For example, trade-offs assume some form of antagonistic pleiotropy, but the reduced performance on saguaro could also result from the accumulation of deleterious mutations in genes involved in adaptation to saguaro, as such mutations would be neutral on Santa Catalina Island where this host is absent. As noted by Schiers [26], trade-offs in host plant adaptation are most definitively demonstrated by selection experiments, e.g. [27], or by examining genetic correlations between fitness on different hosts, e.g. [28], both of which could be investigated by future experiments in the *D. mettleri* study system.

While we found evidence of local adaptation for performance, evidence of local adaptation for preference was not as strong. In fact, females from all populations were equally willing to oviposit on saguaro, and thus there was no evidence for local adaptation in the Superstition or Guaymas populations that typically specialize on this host. Although Santa Catalina Island females were, in general, more willing to oviposit on prickly pear than other populations, acceptance of some host species was still low. For example, only 19% island of females laid eggs on tulip prickly pear, despite the fact that larvae had the highest performance on this host. This apparent mismatch between preference and performance is somewhat surprising, but could be explained in at least two ways. One possibility is that the evolution of preference lags behind the evolution of performance adaptations. This explanation is particularly plausible in light of the fact that prickly pear is the only available host on Santa Catalina Island. Another possibility is that flies on Santa Catalina Island may actually prefer other prickly pear hosts. For logistical reasons, we were unable to include the most common prickly pear species on the island, *O. littoralis*, in our experiments. Given its abundance, and the fact that *D. mettleri* has been collected from *O. littoralis* rots (S. Castrezana, personal observation), future studies with this species are necessary to determine whether flies from Santa Catalina Island also are uniquely capable of using this host as a breeding substrate, and whether female preference for this host is actually higher than that observed for tulip prickly pear.

A reduction in gene flow, coupled with adaptation to a novel habitat, can lead to the evolution of reproductive isolating barriers as a byproduct of the adaptive process [29]. Although such conditions exist for the *D. mettleri* population on Santa Catalina Island, there is currently no strong evidence for sexual isolation or intrinsic postzygotic isolation (male sterility) between Santa Catalina Island flies and flies from other populations [14], [30]. However, our results suggest that the possibility of extrinsic postzygotic isolation, which results when hybrids have an intermediate phenotype that falls between parental ecological niches, should be examined [31]. This type of isolation is expected when parental populations are adapted to divergent environments, and adaptation to one environment is accompanied reduced performance in the alternative environment, as we observed for *D. mettleri* populations.

### Preference/performance on non-natural hosts

Despite evidence consistent with local adaptation and trade-offs, *D. mettleri* appears to be capable of using a broad range of host plant resources, as preference/performance combinations were relatively high on a number of different host species. Interestingly, taxonomic relationships and chemical similarity between host plants were not always predictive of relative levels of preference or performance on different hosts. For example, organ pipe and agria are closely related and similar chemically [16], [17], but all females rejected agria as an oviposition substrate while females from some populations accepted organ pipe at relatively high levels. Similarly, flies within populations also often showed divergent performance on these hosts. The fact that preference/performance combinations were reasonably high for a number of plants that rarely serve as hosts in nature suggests that factors other than plant chemistry also influence host plant associations in mainland/Baja populations. The most likely explanation is that many of the hosts are smaller than saguaro and cardón, and thus rarely produce enough rot exudates to soak soil for an extended period of time. Indeed, *D. mettleri* has been reared from organ pipe soaked soil on rare occasions where rots are large enough, indicating that infrequent associations with this, and other hosts, might be explained lack of opportunity rather than physiological limitations [11].

Overall, our results suggest that ecological opportunity and adaptation to host plant chemistry are important factors contributing to diversity and niche breadth in geographically separated *D. mettleri* populations. While host plant chemistry is clearly important on its own, the microorganisms (bacteria and yeasts) that are associated with cactus rots also may influence patterns of association through their effects on the chemical composition of rots [16], [32]–[34]. This adds an interesting layer of complexity to our study system, and future experiments that incorporate manipulation of microorganismal communities will provide further insights into the evolution of host plant associations in the *D. mettleri* study system.

## Supporting Information

Figure S1 **Eggs laid on natural and non-natural hosts.** Least square mean comparisons of eggs laid on (a) natural and (b) non-natural hosts. In (a) natural hosts for each population are given in parentheses in the key. In (b) locations in which each cactus species is available are given at the bottom (G = Guaymas; S = Superstition; L = Loreto; C = Catalina). For a given host plant, points under different letters were significantly different after Tukey's adjustment (α = 0.05). Points with no letter were not included in the statistical analysis (see methods).
(TIF)

Figure S2 **Host acceptance on natural and non-natural hosts for each population.** Least square mean comparisons of acceptance of different cactus species within populations. The natural host for each population is given in parentheses at the bottom. For a given host plant, points under different letters were significantly different after Tukey's adjustment (α = 0.05). Points with no letter were not included in the statistical analysis (see methods).
(TIF)

Figure S3 **Larval performance on natural and non-natural hosts for each population.** Least square mean comparisons of larval survival on different cactus species within populations. The natural host for each population is given in parentheses at the bottom. For a given host plant, points under different letters were significantly different after Tukey's adjustment (α = 0.05).
(TIF)
