# Supplementary figures and images for: Host Plant Adaptation in Drosophila mettleri Populations
Source: PLoS One. 2012 Apr 6;7(4):e34008. doi: 10.1371/journal.pone.0034008 (PMC3320901; doi:10.1371/journal.pone.0034008)

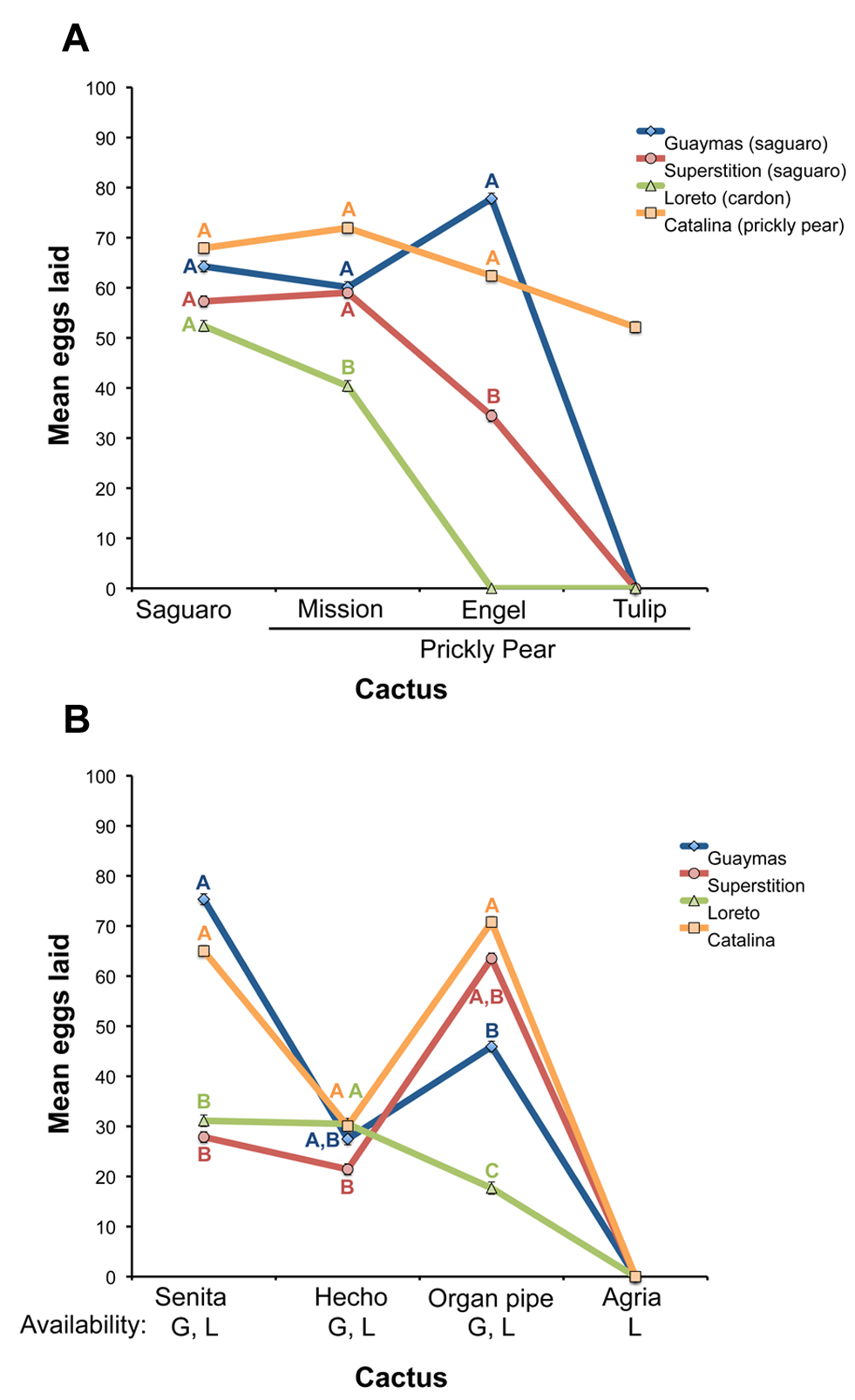

Supplement: Figure S1 — Eggs laid on natural and non-natural hosts. Least square mean comparisons of eggs laid on (a) natural and (b) non-natural hosts. In (a) natural hosts for each population are given in parentheses in the key. In (b) locations in which each cactus species is available are given at the bottom (G = Guaymas; S = Superstition; L = Loreto; C = Catalina). For a given host plant, points under different letters were significantly different after Tukey's adjustment (α = 0.05). Points with no letter were not included in the statistical analysis (see methods). (TIF) [file pone.0034008.s001.tif]

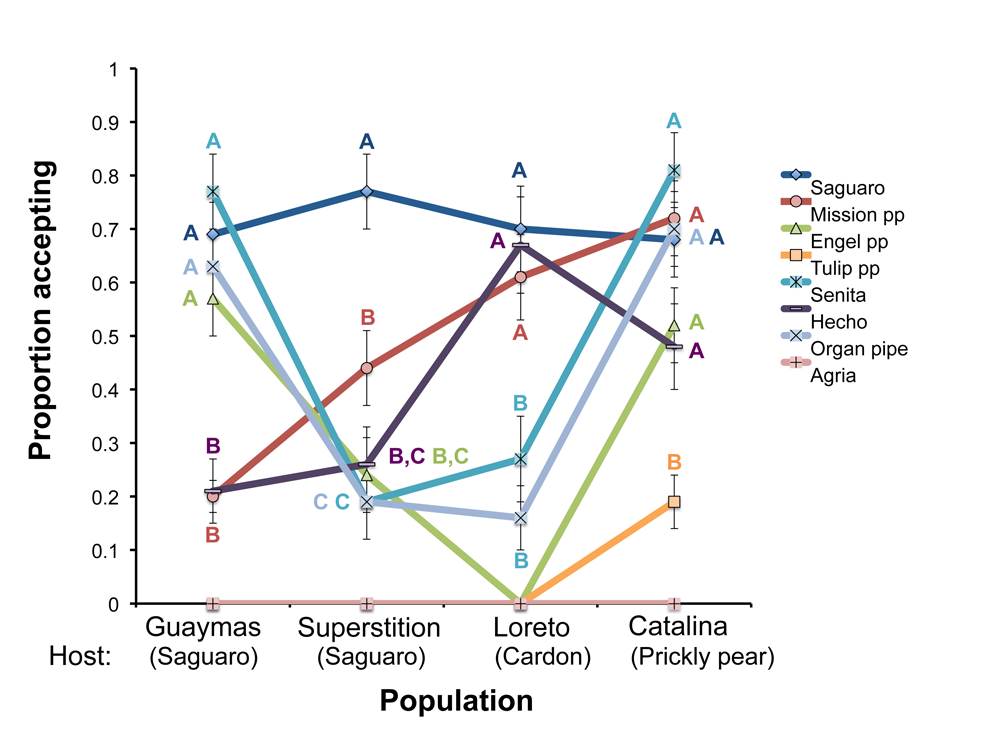

Supplement: Figure S2 — Host acceptance on natural and non-natural hosts for each population. Least square mean comparisons of acceptance of different cactus species within populations. The natural host for each population is given in parentheses at the bottom. For a given host plant, points under different letters were significantly different after Tukey's adjustment (α = 0.05). Points with no letter were not included in the statistical analysis (see methods). (TIF) [file pone.0034008.s002.tif]

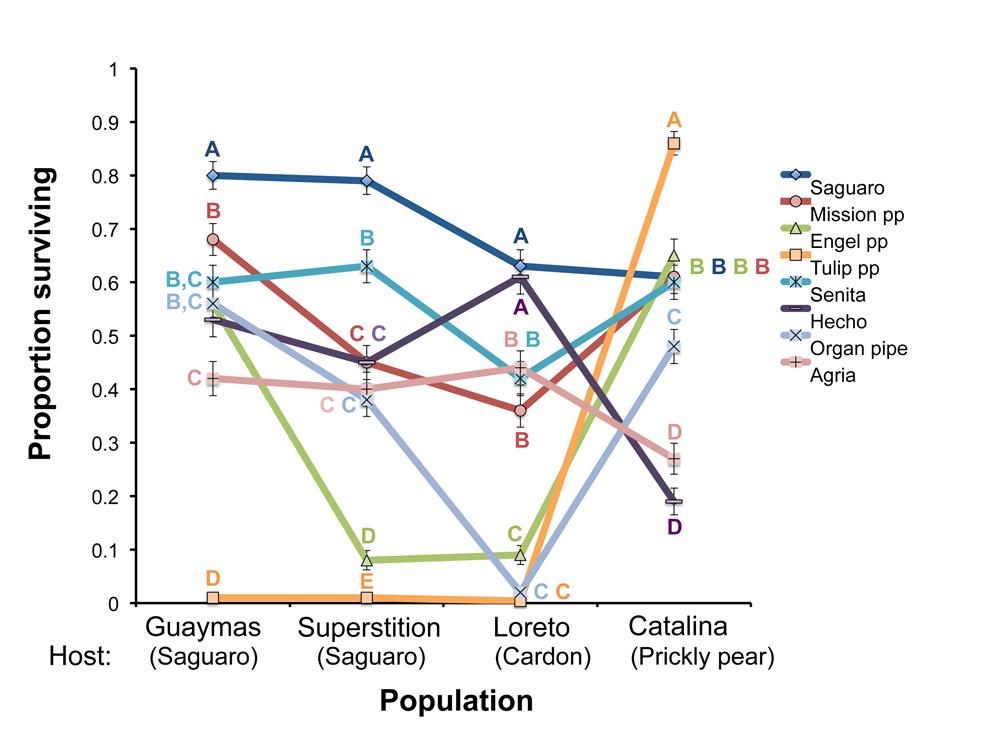

Supplement: Figure S3 — Larval performance on natural and non-natural hosts for each population. Least square mean comparisons of larval survival on different cactus species within populations. The natural host for each population is given in parentheses at the bottom. For a given host plant, points under different letters were significantly different after Tukey's adjustment (α = 0.05). (TIF) [file pone.0034008.s003.tif]
